# Case report: Histiocytic sarcoma as transdifferentiation of a marginal zone lymphoma—a case presentation based on *post mortem* examination and review of the literature

**DOI:** 10.3389/fonc.2025.1609694

**Published:** 2025-09-17

**Authors:** Annaïse J. Jauch, Ilaria Alborelli, Ilaria Balestri, Fatime Krasniqi, Alexandar Tzankov, Benjamin Kasenda, Thomas Menter

**Affiliations:** ^1^ Division of Hematology, University Hospital Basel, Basel, Switzerland; ^2^ Institute of Medical Genetics & Pathology, Pathology, University Hospital Basel, Basel, Switzerland; ^3^ Division of Medical Oncology, University Hospital of Basel, Basel, Switzerland

**Keywords:** marginal zone lymphoma, histiocytic sarcoma, rare disease, autopsy, NGS - next generation sequencing, IGH sequencing

## Abstract

**Background:**

Histiocytic sarcoma (HS) is a rare and aggressive hematopoietic malignancy characterized by the proliferation of cells resembling mature histiocytes. It typically presents in extranodal sites such as the skin, the gastrointestinal tract, and soft tissues and is often accompanied by systemic symptoms including fever and weight loss. HS occurs *de novo* or results from transformation/transdifferentiation from other hematological neoplasms, such as low-grade B-cell lymphomas. To date, only four cases of HS arising from marginal zone lymphomas (MZL) have been documented.

**Case presentation:**

We describe a 66-year-old female patient who presented primarily with abdominal pain and constitutional symptoms. The clinical evaluation showed significant hepatosplenomegaly and lymphadenopathy. A liver biopsy demonstrated a sinus-associated spread of HS. The patient died of suspected hemorrhagic shock before the diagnostic results were finalized and before rescue treatment could be initiated. The autopsy findings confirmed a widespread metastatic HS and concurrent MZL. The molecular analysis showed that both neoplasmas were clonally related, supporting the hypothesis of transformation/transdifferentiation of MZL into HS.

**Conclusion:**

We have reported the fifth unusual transformation of a MZL into a HS. Transformed/transdifferentiated HS is a rare and aggressive neoplasm. Evidence from the published case reports suggests that its clinical course may be more severe than *de novo* HS. This underscores the importance of investigating rare presentations and considering the possibility of an underlying pre-existing hematological malignancy.

## Introduction

Histiocytic sarcoma (HS) is a hematopoietic malignancy characterized by the proliferation and accumulation of neoplastic cells with macrophage-like features and is associated with an aggressive clinical course (1). HS is classified as a subtype of histiocytosis or histiocytic/dendritic cell neoplasms (1). HS can infiltrate virtually any organ, including bone, skin, soft tissues, gastrointestinal tract, and central nervous system (2). Typically, the neoplastic cells exhibit a discohesive growth pattern and sinusoidal distribution, particularly when involving the lymph nodes, liver, and spleen. The malignant cells are predominantly large and oval-shaped, although spindle-cell variants may occasionally occur. They contain abundant eosinophilic cytoplasm and display distinct oval to indented nuclei with vesicular chromatin patterns and prominent nucleoli. The remarkable feature of this malignancy is the consistent presence of an inflammatory infiltrate, which contributes significantly to the tumor’s microenvironment (3). Between 30% and 50% of HS cases are believed to arise from secondary transformation/transdifferentiation or pre-existing hematological malignancies. The best-documented transformations involve low-grade B-cell lymphomas, including follicular lymphoma (4, 5) and chronic lymphocytic leukemia/small lymphocytic lymphoma (CLL/SLL) (6). Transformation rarely occurs from more aggressive neoplasms, such as acute lymphoblastic leukemia (ALL) (7) or mediastinal germ cell tumor (8). HS is only rarely observed in the context of myeloid malignancies (reviewed in Faria and Tzankov (9)). To date, five cases—including our own—describe the transformation/transdifferentiation of a marginal zone lymphoma (MZL) to HS (**Table 1**) (10–13). Transdifferentiation refers to a cellular process in which a cell undergoes a change in lineage identity. Three proposed models explain this process: (a) evolution from a common progenitor, (b) direct transdifferentiation, and (c) dedifferentiation followed by re-differentiation (model derived primarily from studies of follicular lymphoma and HS) (4, 5, 14–16). Although the term “transformation/transdifferentiation” is commonly used in the context of HS arising from low-grade B-cell lymphomas, molecular studies of several cases may actually reflect divergent differentiation from a common progenitor cell rather than true transdifferentiation (4, 17–20).

**Table 1:** Comparison of all cases on histiocytic sarcoma related to marginal zone lymphoma.

Patients	Age, sex	Primary site of MZL	Primary site of HS	Clonal relatedness and mutations	Therapy	Outcome
*Our case*	66 years old, female	Liver, bone marrow, lymph nodes	Spleen, liver, abdominal lymph nodes, kidneys	Analyzed via *IGH* fragment analysis *BRAF^v600E^* Not mutated	None, due to rapid evolution but was planned	Death due to hemorrhagic shock
*Álvaro et al. (* 10 *)*	52 years old, female	Stomach, spleen	Stomach, spleen	Not assessed	Gastrectomy, CHOP	Complete remission after 18 months
*Vaughn et al. (* 11 *)*	63 years old, female	Spleen	Skull base, bone marrow	Not assessed. *BRAF^v600E^* mutated	None, due to rapid evolution but was planned	Disseminated intravascular coagulation, non-cardiac pulmonary edema, acute hypoxemic respiratory failure
*Sabatini et al. (* 12 *)*	53 years old, female	Lymph nodes	Paracervical spinal mass infiltrating paravertebral muscles and neural foramina	CDR3 junction analysis of *IGH*, NGS	Bendamustine, rituximab, R-CHOP (before HS diagnosis)	na
*Komata et al. (* 13 *)*	64 years old, male	Spleen, pancreatic mass	Pancreas and other organs (not specified)	*IGH* and exome sequencing	Bendamustine, rituximab	Death due to acute renal failure

CDR3, complementary determining region 3; HS, histiocytic sarcoma; MZL, marginal zone lymphoma; n.a., not available; NGS, next-generation sequencing; R-CHOP, rituximab cyclophosphamide doxorubicin vincristine prednisone; y, years.

HS frequently harbors somatic mutations in genes involved in the canonical mitogen-activated protein kinase (MAPK) pathway (e.g., *BRAF, NF1, MAP2K1*, *NRAS*, and *KRAS*) and PI3K-AT signaling pathway, with variable frequencies (19). Among these, the most common *BRAF* mutation involves the p.V600E substitution (21). Notably, the acquisition of mutations in these signaling pathways also appears to be decisive in the transformation of low-grade lymphomas into HS (4, 17–20).

Beyond conventional chemotherapy, novel therapeutic strategies targeting the MAPK pathway are being explored, including BRAF inhibitors (e.g., vemurafenib, dabrafenib) and MEK inhibitors (e.g., trametinib, cobimetinib).

## Case description

Here we present the case of a 66-year-old woman who initially presented with abdominal pain. While on holiday in Turkey, she asked for medical assistance at a nearby hospital and was treated for presumed constipation. However, her abdominal symptoms progressively worsened over the following 2 weeks, prompting an early return to Switzerland, where she subsequently consulted her family physician. She reported a single episode of vomiting; there was no fever but drenching night sweats and profound fatigue. The initial laboratory investigations documented a severe inflammatory process, prompting referral to our emergency department. Her medical history included hypothyroidism, arterial hypertension, and type II diabetes. Besides her recent travel to Turkey, she had been residing in Switzerland in the preceding months.

The initial laboratory tests showed normocytic hypochromic anemia, marked thrombocytopenia (21 × 10 (9)/L), acute renal failure, elevated cholestatic and hepatic enzymes, increased lactate dehydrogenase (LDH), and elevated inflammation markers (**Table 2**).

**Table 2:** Hematological parameters in the peripheral blood.

			Patient	
Analysis	Unit	Reference	59 years old, healthy	66 years old, with HS
Hemoglobin	g/L	120–160	146	82
Reticulocytes	×10^9^/L	40–140	na	164
Thrombocytes	×10^9^/L	150–450	194	21
WBC	Leukocytes	4.5–10 × 10^9^/L	6.41	9.75
	Neutrophils	1.3–6.7 × 10^9^/L	3.75	8.02
	Monocytes	0.12–0.62 × 10^9^/L	0.167	0.35
	Eosinophils	<0.3 × 10^9^/L	0.115	0.12
	Basophils	<0.09 × 10^9^/L	0.045	0.02
	Lymphocytes	0.9–3.3 × 10^9^/L	na	0.86
				
Coagulation	INR	<1.3	1	1.2
	aPTT	23–33 s	na	32
	Thrombin time	16–25 s	na	22
	Fibrinogen	g/L	na	2.2
Electrolytes				
Lactate	mmol/L	<2.2	2.1	5.1
Sodium	mmol/L	136–145	140	132
Potassium	mmol/L	3.4–4.5	3.8	4.9
Chloride	mmol/L	98–107	102	95
Calcium albumin corrected	mmol/L	2.1–2.65	2.44	2.5
Phosphate	mmol/L	0.8–1.5	1.42	1.59
Kidney				
creatinine	µmol/L	45–84	55	83
eGFR	mL/min/1.7		98	63
Urea	mmol/L	3–7.8	4.7	13.7
Uric acid	µmol/L	173–359	na	385
Liver				
Bilirubin	µmol/L	<15	7	29.1
AST	U/L	11–34	34	80
ALT	U/L	8–41	44	58
gGT	U/L	178	27	178
AP	U/L	35–105	86	313
Others				
LDH	U/L	135–214		448
CRP	mg/L	<10	3	129.2
Procalcitonin	µg/L	<0.1	na	2.52
Total proteins	g/L	64–83	70	45
Albumin	g/L	35–52	35	18

AP, alkaline phosphatase; AST, aspartate aminotransferase; ALT, alanine aminotransferase; CRP, C-reactive protein; eGFR, estimated glomerular filtration rate; gGT, gamma-glutamyltransferase.

**Figure 1:**
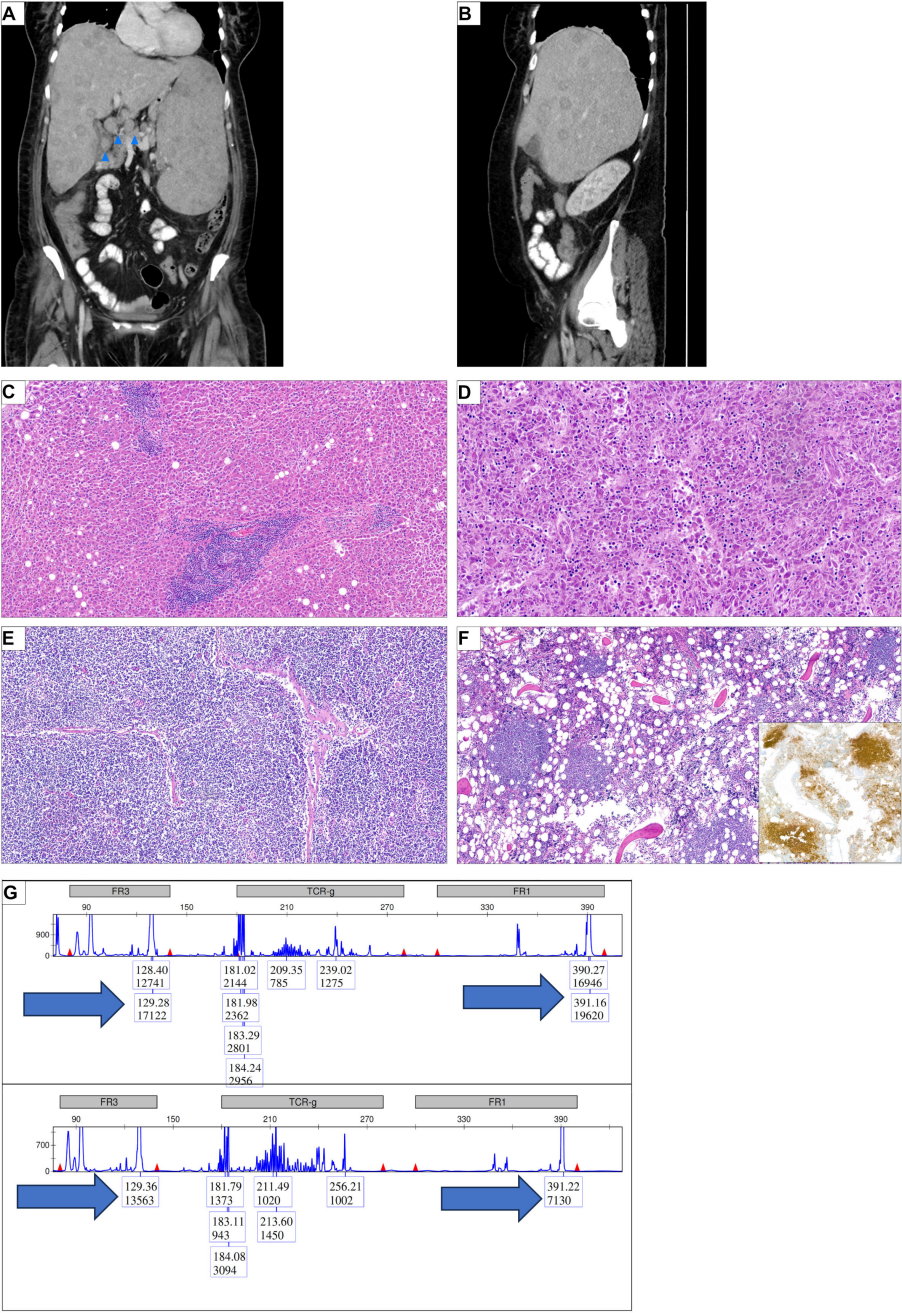
Diffuse abdominal organ infiltration of a histiocytic sarcoma and a marginal zone lymphoma. **(A)** Initial computer tomography (CT) scan of the abdomen, coronal view. Significant hepatosplenomegaly (each 22 cm); diffuse nodular infiltration in the spleen and liver. Blue arrows indicate lymphadenopathy, here exemplified at the liver hilus. **(B)** Sagittal view of the abdominal CT scan; seen is a pronounced hepatomegaly with nodular infiltration. **(C)** Liver section showing focal infiltrates of small mature lymphoid cells representing infiltrates of the marginal zone lymphoma (HE, ×100). **(D)** Liver section showing a dense infiltrate of pleomorphic tumor cells which could be characterized as histiocytic sarcoma (HE, ×200). **(E)** Lymph node section showing diffuse infiltrates of the marginal zone lymphoma (HE, ×100). **(F)** Bone marrow showing nodular infiltrates of lymphoid cells which could be characterized as B-cells in the immunohistochemical stain for CD20 (insert) (HE ×100; IHC ×100). **(G)** Multiplex PCR in combination with high-resolution fragment analysis of the *IGH* locus was performed on autopsy samples showing either involvement by the marginal zone lymphoma or the histiocytic sarcoma alone. In both framework region 1 (FR1) and FR3 regions, the same fragment length could be observed (arrows).

A contrast-enhanced computer tomography (CT) scan was performed to locate the source of inflammation. Imaging showed massive hepatosplenomegaly (liver and spleen both measuring 22 cm) including multiple diffuse lesions in both organs (**Figures 1A, B**). Pancreatic lesions were observed. Additionally, significant intra-abdominal lymphadenopathy was noted (up to 2.5 cm), being most pronounced at the liver hilum, retroperitoneal, and interaortocaval regions (**Figure 1A**, blue arrows for adenopathy at the liver hilum). A diffusely metastasizing malignancy was suspected, and the patient was admitted to the internal medicine ward for further evaluation. Active systemic infections with human immunodeficiency virus (HIV) and hepatitis B and C virus (HBV and HCV, respectively) were excluded.

A liver biopsy was performed with platelet transfusion support. Conventional histopathology revealed a sinus-associated liver infiltration by a discohesive, mature large-blastoid (immunoblastoid) neoplasm suggestive of high-grade lymphoma. We therefore planned to treat the patient with CHOP chemotherapy containing cyclophosphamide, doxorubicin, vincristine, and prednisone despite pending definite histopathological confirmation.

The night prior to initiating chemotherapy, the patient received prophylactic hydration, dexamethasone, and rasburicase (a recombinant form of urate-oxidase enzyme used to manage hyperuricemia). That evening, the patient developed restlessness and subjective dyspnea. The symptoms stabilized for the next 2 h with 3 L of oxygen per mask. However, the dyspnea worsened, and additionally, she developed tachycardia (103 bpm) and tachypnea (30/min), and her oxygen saturation dropped to SpO_2_ 88% despite 4 L of oxygen. A blood gas analysis revealed a hemoglobin level of 60 g/L (down from 90 g/L that morning) and severe metabolic acidosis (lactate 14 mmol/L, pH 7.0), consistent with hemorrhagic shock. While being transferred to the intensive care unit (ICU), the patient became bradycardic until no pulse was palpable. Cardiac monitoring showed a pulseless electrical activity. She immediately received cardiopulmonary resuscitation, including epinephrine, followed by atropine. After 35 min of unsuccessful reanimation, we decided to terminate the resuscitation.

The patient’s relatives consented to a *post-mortem* examination. The autopsy confirmed the suspicion of an advanced metastatic neoplasm involving the liver, spleen, kidneys, and intra-abdominal lymph nodes. The immunohistochemistry demonstrated that the neoplastic cells expressed histiocytic antigens such as CD11c, CD14, CD68, and PU.1 and were negative for lymphoid and endothelial markers. Additionally, no expression for CD1a, IRF8, Langerin, lysozyme, and S100 was observed. The proliferation index was 60% (assessed with an antibody against MIB-1). The results of further stainings against CD34, CD117, and TdT were negative. These findings confirmed the diagnosis of histiocytic sarcoma (HS). Notably, the tumor cells were negative for the BRAF mutation p.V600E and showed no overexpression of pERK.

In parallel, nodular lymphoid aggregates composed of CD20^+^ and BCL-2^+^ B-cells with a so-called follicular colonization pattern were identified in the liver, the bone marrow, and the lymph nodes (**Figures 1C-F**). These B-cells lacked light chain restriction. A rearrangement for *BCL-2* was not found in a break-apart probe, and the cells were negative for BCL-6, MEF2B, CD10, MUM1, and EBV (EBER). This profile was consistent with marginal zone lymphoma (MZL).

Given the frequent co-occurrence of HS with hematological malignancies, we investigated clonal relatedness between MZL and HS. A polymerase chain reaction (PCR) analysis of the immunoglobulin heavy chain (IGH) framework region 3 (FR3 region) revealed identical fragment length in both tumors (**Figure 1G**). To investigate further the degree of clonal relatedness between the two tumors, we characterized the mutational landscape with next-generation sequencing (NGS). We observed that both tumors shared four mutations, two nonsense mutation *NOTCH2* and *CDKN2A*, as well as the missense mutations in *CARD11* (**Table 3**). Paralleling the aggressiveness of the HS, the variant allele frequency (VAF) was more elevated in the HS than in the MZL. Additionally, one might hypothesize that the MZL transformation was accelerated by acquiring an additional gain of function mutation in *MAP2K1* (**Table 3**). The clonality assay and the NGS suggest a clonal relationship and consistent with transformation/transdifferentiation from MZL to HS.

**Table 3:** Next-generation sequencing experiment regarding the mutational landscape of marginal zone lymphoma and histiocytic sarcoma.

Gene	Protein change	Variant classification	Pathogenicity	MZL	HS
*NOTCH2*	p.Arg2400Ter c.7198C>T	Nonsense mutation	Pathogenic/likely pathogenic	9%	38%
*NOTCH2*	p.Ser2145Ter c.6434C>G	Nonsense mutation	VUS	12%	42%
*CARD11*	p.Tyr361Cys c.1082A>G	Missense mutation	Pathogenic/likely pathogenic	15%	44%
*CDKN2A*	p.Arg80Ter c.238C>T	Nonsense mutation	Pathogenic/likely pathogenic	17%	44%
*MAP2K1*	p.Lys57Asn c.171G>T	Missense mutation	Pathogenic/likely pathogenic	0%	18%

The percentage values refer to the variant allele frequency.

Asn, asparagine; Arg, arginine; Cys, cysteine; HS, histiocytic sarcoma; MZL, marginal zone lymphoma; Ser, serine; Ter, terminal.

## Discussion

In this case study, the rapid decline in the patient’s overall health indicated the presence of an aggressive underlying pathology, necessitating imaging studies and a biopsy. Despite recognition and an initial plan for cytoreductive therapy, the patient died due to hemorrhagic shock before treatment could be initiated. The *post-mortem* examination identified widespread HS co-existing with clonally related MZL, suggesting transformation/transdifferentiation of an indolent lymphoma into an aggressive histiocytic neoplasm.

HS is a rare histiocytic/dendritic cell malignancy (1) characterized by heterogeneous clinical manifestations that span from localized lesions affecting single anatomic sites to an aggressive systemic disease (22). The site affected involve mostly connective tissue/skin, followed by the respiratory tract and gastrointestinal system (22). Treatment strategies are determined by disease extent, specifically distinguishing between unifocal and multisystem involvement. A unifocal disease may profit from involved field radiation and/or surgery (23–25) and are associated with a slower progress and a better overall survival (hazard ratio 0.33) (22). For a disseminated disease, multiagent chemotherapy regimens are typically employed. The most frequently utilized protocols include ICE (ifosfamide, carboplatin, and etoposide) or CHOP. In a few cases, a consolidation with autologous stem cell transplantation was performed (26); however, these regimens have not undergone a direct comparison in prospective clinical trials.

To better understand the rare event of MZL transforming/transdifferentiating into HS, we reviewed the literature for all descriptions and identified four previously reported cases (**Table 1**). The mean age at presentation was 59.6 years (range 52–66 years), with a female predominance (4/5 cases). In contrast, primary HS presents at a younger median age (51 years) and shows a slight male predominance (27). The MZL localization varied, ranging from lymphatic to non-lymphatic tissues (**Table 1**). The MZL-transformed/transdifferentiated HS were multilocular in all patients, affecting in all patients at least two different anatomical sites (**Table 1**). In primary HS, a multilocular disease is associated with poorer prognosis (28).

Vaughn et al. proposed that *BRAF^V600E^* may act as a genetic driver in MZL transforming/transdifferentiating into HS and the aggressive nature of the disease (11). Although our patient tested negative for *BRAF^V600E^*, we observed downstream of *BRAF* a gain of function mutation in *MAP2K1.* Future research will clarify the molecular events that are key to the evolution of transformed/transdifferentiated HS.

One cornerstone of characterizing such transformation/transdifferentiation is demonstrating clonal relatedness of the different neoplasms. This can be achieved in various ways. The most robust approach in this context is PCR-based fragment length analysis available for more than 30 years PMID (29). Further sophisticated methods include high-throughput sequencing-based approach to search for pathogenic mutations and/or clonality. After having proven the clonal relationship between the MZL and the HS, we could also prove the clonal relationship (four common pathogenic mutations) as well as progression of the HS by acquiring a pathogenic *MAP2K1* mutation.

In three of five reported cases (including ours), a molecular analysis of the *IGH* locus confirmed a shared clonal origin (**Table 1**). We used multiplex PCR followed by high-resolution *IGH* fragment analysis, Sabatini et al. (12) applied next-generation sequencing (NGS) of the *IGH* complementary determining region 3 (CDR3), and finally Komata et al. (13) utilized *IGH* locus reconstitution. The remaining two reports lacked molecular confirmation (10, 11). In light with previous research on indolent lymphomas developing into HS (5, 17, 19, 30), we interpreted the clonal relationship as either transformation/transdifferentiation from MZL into a widespread HS or alternatively the divergent differentiation of both malignancies from a common progenitor, as previously shown for a follicular lymphoma (4).

Interestingly, Komata et al. (13) described a case with autoimmune hemolytic anemia (AIHA) (13). Retrospectively, our patient’s bicytopenia may have reflected a paraneoplastic Evans syndrome (sequential or concomitant appearance of AIHA and immune thrombocytopenia), a rare manifestation associated with hematologic malignancies (31).

In all five patients, chemo-/immunotherapy was scheduled (**Table 1**). One patient with localized splenic and gastric MZL/HS manifestation underwent gastrectomy and adjuvant chemotherapy (CHOP protocol) (10), being in complete remission after 1.5 years (10). Two patients received bendamustine and rituximab, and one outcome was not reported (12), while the second patient died due to acute renal failure (13). Similar to our case description, one patient died before treatment initiation due to rapid disease progression (11). Vaughn et al. described death from disseminated intravascular coagulation, pulmonary edema, and consequently hypoxemic respiratory failure (11). The latter emphasizes the need for additional research to determine the ideal treatment combination for transdifferentiated/transformed HS to optimize patient outcomes and quality of patient life.

Understanding the transdifferentiation pattern in different lymphomas provides key insights into clonal evolution. The more cases that are studied, the better the biology behind transformation/transdifferentiation will be understood. Cumulative evidence from the reported cases suggests that HS with MZL may run a more dismal clinical course than primary HS; thus, an underlying hematological neoplasm in any HS has to be ruled out for correct risk stratification.

## Materials and methods

### Ethics approval and consent to participate

The patient was enrolled in this study after providing informed consent, which was discussed upon her admission to the hospital. The study received approval from the Ethics Committee of Northwestern and Central Switzerland, ensuring compliance with all relevant national and international ethical standards.

### Radiology and laboratory blood analysis

All available radiological examinations and blood tests for the patient were reviewed and conducted as part of the standard clinical routine.

### Histological specimens, immunohistochemical staining

Formalin-fixed, paraffin-embedded (FFPE) tissue specimens were sectioned at a thickness of 4 μm and mounted onto adhesive-coated slides. Immunohistochemical staining was subsequently performed using an automated staining system.

### Fragment length IGH analysis and next-generation sequencing

The immunoglobulin heavy chain (*IGH*) gene rearrangement gene products were amplified using consensus FR3 and J primers, as published earlier (29, 32). PCR products were run on a high-resolution fragment length analyser (ABI 310 Genetic Analyzer, Applied Biosystems). The amplicons were assessed by capillary electrophoresis and laser-induced fluorescence detection. Procedures followed the manufacturer’s recommendations.

From formalin-fixed paraffine-embedded (FFPE) samples total DNA was extracted and utilized for high-throughput sequencing of the FR3-J region of *IGH*, and of the immunoglobulin lights chains *IGK*/*IGL*, as well as KDE and Cint-containing rearrangements. The libraries were made using 200 ng total DNA input in a single-pool multiplex PCR with the Oncomine BCR Pan-Clonality Assay (Thermo Fisher Scientific, Waltham, US)., according to manufacturers’ instructions. Libraries were quantified and diluted to 50 pmol final concentration. Sequencing was performed on an Ion GeneStudio S5 Prime instrument (Thermo Fisher Scientific, Waltham, US).

Both the PCR- and the NGS-assay are intended for use of FFPE tissue and are also currently used in the routine diagnostic setting. Quality controls accompanying both assays proved the validity of the results.

### Data analysis and illustrations

The GeneMapper Software (Thermo Fisher Scientific, Waltham, US) was used for fragment analysis. The Ion Reporter Software v5.18 (Thermo Fisher Scientific, Waltham, US) was used for analysis of the sequencing output generated using the Oncomine BCR Pan-Clonality Assay Figure were generated with Affinity Designer (v.1.10).

## Data Availability

Any data are available from the corresponding authors upon request.
